# Comment on “Alternate Sequential Suture Tightening: A Novel Technique for Uncontrolled Postpartum Hemorrhage”

**DOI:** 10.1155/2015/279513

**Published:** 2015-07-21

**Authors:** Shigeki Matsubara, Hironori Takahashi, Alan K. Lefor

**Affiliations:** ^1^Department of Obstetrics and Gynecology, Jichi Medical University, Tochigi 329-0498, Japan; ^2^Department of Surgery, Jichi Medical University, Tochigi 329-0498, Japan

We read with interest the recent article “Alternate Sequential Suture Tightening: A Novel Technique for Uncontrolled Postpartum Hemorrhage” by Ghosh and Mala [1]. They developed a novel technique of uterine compression suture (UCS) for atonic bleeding where a Hayman suture [2] is performed, but importantly they sequentially tighten the knot, yielding “very tight” compression. In 92% (11/12) of their patients, the UCS alone stopped the bleeding. No patients had untoward sequelae. Their data is promising; however, we have two concerns.

First, we wonder if such a tight knot is needed. In the Ghosh technique, the final knot was eventually tightened compared to the initial knot by as much as 3–5 cm, meaning tighter by 3–5 cm compared with an ordinary Hayman suture [2]. In our opinion, the Ghosh suture may be too tight and we wish to describe two supporting pieces of evidence. First, we usually use the Matsubara-Yano (MY) UCS in patients with atonic bleeding (Figure 1(a)) [3] and sometimes even cut the sutures because the knot looks “too tight.” We then reperform the MY UCS, making a looser knot. A knot that is too tight may lead to tissue damage caudal to the suture, that is, the lower uterine segment. This portion would become thin and weak, and a knot that is too tight would forcefully pull this portion in the cephalad direction, easily damaging this area (Figure 1(b)). We sometimes loosen the knot also for fear of possible subsequent uterine ischemia. After placing the UCS and closing the hysterotomy incision, it is our experience that the uterus becomes, more or less, contracted even in patients with an atonic uterus. Uterine contraction may make the knot even tighter. Second, we are concerned about Figure 1(d) in Ghosh and Mala's article [1]. The uterus looks ischemic in the area between two sutures even 1.5 years after the Ghosh UCS, possibly indicating that the suture significantly deprived blood flow to the uterus. Mowat et al. [4] reported a case of uterine necrosis after B-Lynch suture, describing, “there was central necrosis of the anterior body and fundus of the uterus between the two limbs of the B-Lynch suture”: this feature was similar to Ghosh and Mala's figure (Figure 1(d)) [1].

As described previously, generally speaking, compression force is related to the ability to induce hemostasis but is also related to adverse events associated with UCS [3]. A tight knot or tight suture, exerting excessive compression, naturally may lead to effective hemostasis at the time but may lead to uterine ischemia afterward, meaning that the hemostatic ability and occurrence of ischemia may have a “trade-off” relationship. Since the incidence rate of uterine ischemia is low [3, 4], the fact that it is not reported by Ghosh and Mala does not entirely rule out its occurrence. We proposed the concept of a removable UCS [5] that has been used clinically [6], removing the compression suture within two days postpartum. Strong compression of the uterus for up to two days postpartum and then removing the suture may be reasonable [5, 6].

Second, since the Ghosh technique is a modified Hayman suture [2], a drawback of Hayman suture [2] and B-Lynch suture [7] persists, that is, “the suture sliding out” (Figure 1(c)). We have previously pointed this out [3] and Hayman wrote, “the suture threatened to slide off the uterine fundus, like braces off a round shouldered man” [2]. This may occur more readily with a tight knot. When the uterus temporarily contracts and the knot becomes tighter, the possibility of “sliding out (off)” becomes higher; there may be no room for the suture other than “sliding out.” Sliding out of the suture makes compression insufficient. Figures 1(b) and 1(c) in Ghosh and Mala's article [1] lead us to consider this possibility.

Obstetric practice has surely changed before and after the introduction of the UCS. We have now obtained a powerful tool, the UCS, against postpartum hemorrhage. Every effort should be made to make the UCS better and, thus, we applaud and respect Drs. Ghosh and Mala. The development of a number of UCS techniques shows that there may be no one best UCS. Further study of the UCS and wide discussion are needed.

## Figures and Tables

**Figure 1:**
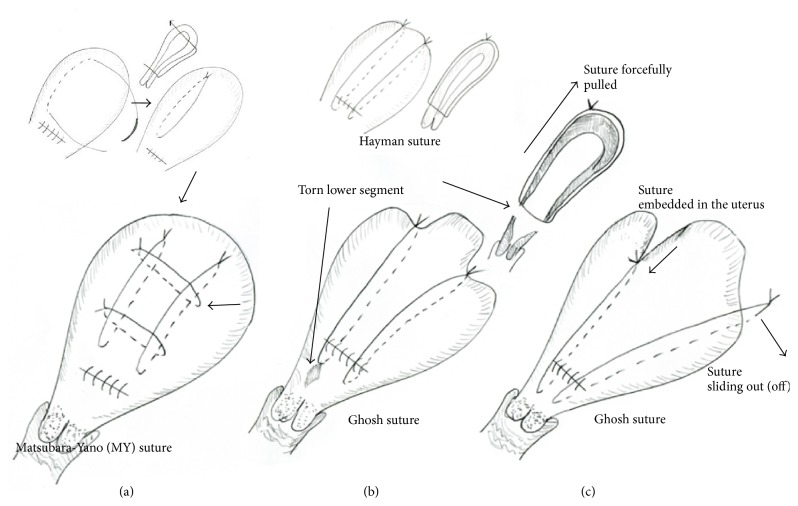
Schematic presentation of the Matsubara-Yano (MY) uterine compression suture (a) and possible drawbacks of the Ghosh suture (b, c). (a) The MY suture consists of two (or three) longitudinal transfixation sutures and two transverse sutures. Upper insets indicate how the first longitudinal transfixation suture is placed. Then, the transverse sutures are placed lateral to the longitudinal suture (arrow), thereby preventing the thread from sliding out (off). (b) Ghosh suture is a modification of Hayman suture (upper left insets). If the knot of a Ghosh suture is too tight, it may destroy the caudal insertion site (arrow), or the lower uterine segment, which is thin and weak. Upper right inset shows the sagittal view. (c) In the Ghosh suture, similar to the B-Lynch or Hayman suture, sutures may “slide out (off)” from the uterine fundus (left), thus yielding insufficient compression. If the knot is too tight, the chance of “sliding out” may be higher, since there may be no room for the suture to move. Even if it does not slide out, the thread may embed in the uterus (right), leading to uterine ischemia.

## Abbreviations

MY: Matsubara-Yano
UCS: Uterine compression suture.

## References

[1] Ghosh S. B., Mala Y. M. (2015). Alternate sequential suture tightening: a novel technique for uncontrolled postpartum hemorrhage. *Obstetrics and Gynecology International*.

[2] Hayman R. G., Arulkumaran S., Steer P. J. (2002). Uterine compression sutures: surgical management of postpartum hemorrhage. *Obstetrics and Gynecology*.

[3] Matsubara S., Yano H., Ohkuchi A., Kuwata T., Usui R., Suzuki M. (2013). Uterine compression sutures for postpartum hemorrhage: an overview. *Acta Obstetricia et Gynecologica Scandinavica*.

[4] Mowat A., Minuzzo L., Wilson J. (2013). A necrotic uterus after a B-Lynch Suture: fertility sparing surgery. *Australian and New Zealand Journal of Obstetrics and Gynaecology*.

[5] Matsubara S. (2014). New prophylaxis methods for adverse events of uterine compression sutures: removing compression threads. *Acta Obstetricia et Gynecologica Scandinavica*.

[6] Zhang Z. W., Liu C. Y., Yu N., Guo W. (2015). Removable uterine compression sutures for postpartum haemorrhage. *BJOG: An International Journal of Obstetrics & Gynaecology*.

[7] B-Lynch C., Coker A., Lawal A. H., Abu J., Cowen M. J. (1997). The B-Lynch surgical technique for the control of massive postpartum haemorrhage: an alternative to hysterectomy? Five cases reported. *British Journal of Obstetrics and Gynaecology*.

